# Gold nanoparticles partition to and increase the activity of glucose-6-phosphatase in a synthetic phospholipid membrane system

**DOI:** 10.1371/journal.pone.0183274

**Published:** 2017-08-17

**Authors:** Tyson J. MacCormack, Amanda M. Rundle, Michael Malek, Abhilash Raveendran, Maria-Victoria Meli

**Affiliations:** Department of Chemistry and Biochemistry, Mount Allison University, Sackville, NB, Canada; Universitetet i Bergen, NORWAY

## Abstract

Engineered nanomaterials can alter the structure and/or function of biological membranes and membrane proteins but the underlying mechanisms remain unclear. We addressed this using a Langmuir phospholipid monolayer containing an active transmembrane protein, glucose-6-phosphatase (G6Pase). Gold nanoparticles (nAu) with varying ligand shell composition and hydrophobicity were synthesized, and their partitioning in the membrane and effects on protein activity characterized. nAu incorporation did not alter the macroscopic properties of the membrane. Atomic force microscopy showed that when co-spread with other components prior to membrane compression, nAu preferentially interacted with G6Pase and each other in a functional group-dependent manner. Under these conditions, all nAu formulations reduced G6Pase aggregation in the membrane, enhancing catalytic activity 5–6 fold. When injected into the subphase beneath pre-compressed monolayers, nAu did not affect G6Pase activity over 60 minutes, implying they were unable to interact with the protein under these conditions. A small but significant quenching of tryptophan fluorescence showed that nAu interacted with G6Pase in aqueous suspension. nAu also significantly reduced the hydrodynamic diameter of G6Pase in aqueous suspension and promoted catalytic activity, likely via a similar mechanism to that observed in co-spread monolayers. Overall, our results show that nAu can incorporate into membranes and associate preferentially with membrane proteins under certain conditions and that partitioning is dependent upon ligand shell chemistry and composition. Once incorporated, nAu can alter the distribution of membrane proteins and indirectly affect their function by improving active site accessibility, or potentially by changing their native structure and distribution in the membrane.

## Introduction

Engineered nanomaterials (ENMs) have unique, size-dependent properties that make them desirable for a range of applications, including drug delivery, diagnostics, catalysts, and more. The incidental release of ENMs through aerosols, terrestrial runoff, and wastewater is projected to increase ENM burdens in the environment [1,2] and toxicity has been noted in a variety of aquatic and marine species [3,4]. Small volume biomedical applications represent little risk to the environment, but the potential for non-target bioactivity has limited their widespread use in therapeutics and imaging [5]. The rapid proliferation of novel ENM formulations and our inability to test each for bioactivity has augmented the need for a better understanding of how ENMs interface with and affect biological systems on a molecular scale [6].

Interactions between ENMs and soluble proteins often leads to the formation of a “protein corona” on the ENM surface, which can facilitate cellular uptake and influence bioactivity [7]. *In vitro*, ENM-protein interactions have also been shown to disrupt the structure and function of soluble proteins [8–10], which may explain *in vivo* toxicity under certain conditions [11]. The physicochemical properties of the ENM and protein dictate if an interaction will occur and what the impact of that association will mean to the structure and function of the protein [12]. Molecular dynamics models indicate that electrostatic forces likely drive these binding events [9,13] and that resulting protein conformation changes can substantially impact ligand binding affinity and catalysis in enzymatic reactions [9]. A large body of work has addressed the mechanisms and consequences of ENM-protein interactions in soluble proteins [7] but much less is known regarding interactions between ENMs and membrane proteins. It is also difficult to accurately assess the effects of hydrophobic ENMs in solution-based systems but these formulations may still interact with membranes and membrane proteins.

In aquatic nanotoxicity studies, membrane proteins appear to be important ENM targets, but the mechanisms by which they are affected remain elusive. In fish, the Na^+^/K^+^-ATPase (NKA) protein lies on the basolateral membrane of the gill epithelium where it plays a critical role in maintaining whole animal ion and acid/base homeostasis. A number of ENMs, particularly metal formulations, potently influence gill NKA activity and their effects cannot always be readily explained by dissolution of bioactive metal ions [14–21]. Changes in protein conformation and ligand binding dynamics resulting from direct interactions with ENMs may explain changes in activity, but membrane proteins are also sensitive to alterations in their surrounding lipid environment. For example; the molecular activity of the NKA protein increases with membrane phospholipid desaturation and resulting increases in lateral pressure in the bilayer core [22]. ENMs can alter the morphology of lipid bilayers or even trigger the formation of pores in membranes [23], and such changes could impact the function of embedded proteins. Characterizing how ENMs interact with membranes and membrane proteins will allow us to better understand the molecular basis of ENM bioactivity and optimize ENM formulations to improve molecular targeting and efficacy in clinical applications and reduce environmental toxicity. It will also aid in understanding how chronic exposure to ENMs may promote misfolding of membrane proteins and increase the risk of developing neurodegenerative diseases like Alzheimer’s [24].

The goal of this study was to characterize how the relative hydrophobicity of gold ENMs (nAu) influence partitioning in a simplified model membrane system and to understand how this may impact the function of an embedded membrane protein. To address this problem, we formed a Langmuir phospholipid monolayer incorporating a catalytically active transmembrane protein, glucose-6-phosphatase (G6Pase). Such membranes are known to accurately mimic the interface between a phospholipid-protein membrane and an aqueous medium and can provide valuable information on how ENMs influence membrane properties [25–27]. Manipulation of these membranes using the Langmuir balance technique enables *in situ* measurement of the membrane response to compression and precise control over their lipid density and surface tension. Coupled with Langmuir-Blodgett transfer to suitable supports for *ex situ* imaging, this approach allows us to characterize how hydrophobic ENMs interact with membrane proteins. Analytical tools that can accurately characterize such mechanisms in the complex membrane environment *in vivo* are lacking but this model system provides a means to extend the knowledgebase on ENM-protein interactions beyond soluble proteins. In the endoplasmic reticular membrane, G6Pase dephosphorylates glucose-6-phosphate (G6P) to glucose and inorganic phosphate in the terminal step of gluconeogenesis. In addition to playing a key role in interprandial glucose homeostasis and the pathophysiology of a number of diseases [28], it is also a potential target for nano-enabled pharmaceuticals. Several ENM formulations exhibit anti-diabetic activity *in vivo* [29,30] and *in vitro* [31] and their effects have been linked to alterations in G6Pase activity or expression.

## Materials and methods

### Reagents and solutions

Reagents used in this study were purchased from Sigma-Aldrich (Oakville, ON, Canada), unless otherwise noted. 1,2-dipalmitoyl-*sn*-glycero-3-phosphocholine (DPPC) was purchased from Avanti Polar Lipids Inc. (Alabaster, AL). D-Glucose-6-Phosphate (G6P) was purchased from Calzyme Laboratories Inc. (San Luis Obispo, CA). The G6Pase preparation was a crude purification from rabbit liver (Sigma-Aldrich) and was approximately 30% protein, with the balance of the mass mostly consisting of sucrose with a small quantity of lipids. SDS-PAGE analysis of the G6Pase preparation (S1 Fig) indicated that the protein was not fully purified, which is expected given the reasons outlined elsewhere [32]. The lipid constituents of the enzyme preparation were characterized by lipid profile analysis as follows. Lipids were extracted using chloroform and methanol in a modified version of the Bligh/Dyer method [33]. A 1.12 mg sample of the isolate was prepared in a solution containing 1 mL chloroform (CHCl_3_), 2 mL methanol (MeOH) and 0.8 mL phosphate buffered saline (1:2:0.8 solution of CHCl_3_/MeOH/saline). This sample was transferred to a glass vial with 25 μL acetic acid (AcOH, 10%), followed by 100 μL 1,2-Diheptadecanoyl-*sn*-glycero-3-phosphorylcholine (0.03 mg mL^-1^, internal standard). Once the extraction was performed, the CHCl_3_ portions were pooled and evaporated followed by base hydrolysis and transmethylation. The resulting solution was split between hexanes and brine; the hexane layer was collected, concentrated under nitrogen gas, and reconstituted in 200 μL hexanes before transfer to gas chromatography vials. Samples were injected and fatty acid methyl esters (FAMEs) were detected by flame ionization. The profile of FAMEs in the G6Pase preparation can be found in S1 Table.

A buffer containing 100 mmol L^-1^ sodium cacodylate trihydrate, pH 6.5, was used for the subphase of the Langmuir-Blodgett trough apparatus and for assessing G6Pase activity in suspension. In monolayer studies, the G6P substrate (8 mmol L^-1^) was added to the subphase immediately prior to use.

### Gold nanoparticle synthesis, purification, and characterization

nAu were prepared and characterized using established methods [34]. Particles were initially coated and stabilized with 4-dimethylaminopyridine (DMAP). This coating was exchanged with thiol in a two-phase exchange to create nAu with varying hydrophobicity [34]. Three batches of nAu were synthesized using this method, characterized by the terminal functional group of thiol ligands: 100% CH_3_ terminated nAu (1-undecanethiol), 100% OH terminated nAu (11-mercapto-1-undecanol), and 25% OH terminated nAu (11-mercapto-1-undecanol and 1-undecanethiol). It should be noted that the 25% OH terminated nAu was previously shown to exhibit improved hydrophilic character (compared with 100% CH_3_ terminated nAu) at the air/water interface, where several studies have indicated non-monotonic trends in hydrophobicity with ligand shell composition [35–37]. nAu were purified by repeated washing cycles of vortex mixing, centrifugation, and separation until ^1^H NMR (200 MHz, Varian Oxford, Palo Alto, CA) showed an absence of unbound alkanethiol. ^1^H NMR was also used to determine the final composition of the mixed ligand shell by I_2_ decomposition using established methods [38]. Transmission electron microscopy (TEM) imaging was used to determine the distribution of nAu core sizes, with samples prepared for analysis as described below.

### Membrane monolayer preparation

The constituents of the *in vitro* membrane system were prepared in one suspension and co-spread on the air-aqueous interface of a Langmuir trough apparatus (KSV Minitrough and Nima 602A film balance; Biolin Scientific, Stockholm, Sweden) which was equipped with a refrigerated and heating water circulator. 500 μL of the 0.2 mol% nAu suspension contained 7.5 mg G6Pase preparation and 0.075 mg DPPC for all trials, with the addition of 0.5 mg nAu for treatment conditions. Dosages in the range of 0.1–0.5% nAu have been shown to have no effect on the monolayer phase behavior seen in the isothermal compression of DPPC membranes, but do have a significant effect on the monolayer structure, particularly the shape and distribution of solid phase lipid in the phase coexistence regime [26]. All components were suspended in a solvent mix of chloroform (CHCl_3_) and dimethyl sulfoxide (DMSO) in a 1:1 ratio. 25–48 μL of this suspension was spread at the air-water interface in a dropwise fashion and the solvent was allowed to evaporate for 20 min. The mixed monolayer was compressed to a physiological pressure of 30 mN m^-1^ [22] at a speed of 10 cm^2^ min^-1^, with surface pressure measured by a filter paper Wilhelmy plate. Full isotherms of 220 μL of spread suspension were obtained in triplicate to monitor potential changes in membrane compressibility or collapse pressure. A Bradford protein assay was performed on the subphase solution and protein was not detected (data not shown), confirming that G6Pase did not partition into the subphase following spreading of the monolayer. The specific activity of G6Pase measured in the monolayer ranged from 20 to 110% of the specific activity in the stock enzyme preparation, depending on the treatment conditions.

### Influence of nAu on membrane G6Pase activity

500 μL samples of subphase were taken from beneath the trough barriers at times 0, 10 and 30 min immediately following compression of the monolayer to 30 mN m^-1^. Samples were assayed for inorganic phosphate (P_i_) using the Taussky-Shorr Color Reagent [39] and compared to a standard curve of 1 to 5 μg mL^-1^ P_i_. The average activity (N = 7 trials, on average) was normalized to the amount of protein loaded on the trough.

Trials were also performed in which nAu were introduced through the subphase following full compression of the synthetic membrane. The monolayer (without nAu) was spread and compressed to 30 mN m^-1^, as described above, and then 500 μL of a 0.1 mg mL^-1^ suspension of nAu in ethanol were injected into the gently stirred subphase. Subphase samples were taken at 30 min prior to spreading (-30 min), halfway through the solvent evaporation phase (time = -20 min), and at 0, 10, 20, 30, 40, and 50 min following introduction of nAu to the subphase.

### Influence of nAu on G6Pase structure and function in suspension

The impact of nAu on the conformation of G6Pase in suspension was qualitatively assessed by examining the intrinsic fluorescence of tryptophan amino acid residues in the protein [40]. The fluorescence of monolayer spreading solution containing DPPC and G6Pase in a 1:1 solvent mixture of DMSO and chloroform, with and without nAu (0.1 mg mL^-1^), was measured at 280/350 nm (ex/em) using a fluorimeter (LS 50B, Perkin Elmer, Waltham, MA). The effect of nAu on G6Pase activity in suspension was also assessed to compare with previous studies on ENM interactions with soluble proteins. G6Pase was prepared in cacodylate buffer with or without 100% OH terminated nAu (0.1 mg mL^-1^ in EtOH) and samples were taken at 0, 5, 10, 15, 20, 25, and 30 minutes immediately following the addition of the G6P substrate. Samples not containing nAu were treated with an equivalent volume of EtOH to control for possible solvent effects. Enzyme activities are presented as mean ± standard error of the mean (SEM) and comparisons between treatments were done using a one-way ANOVA with Tukey’s post hoc tests for individual comparisons (GraphPad Prism, Version 5.0).

### Atomic force microscopy imaging

Atomic force microscopy (AFM) was used to characterize the surface topography of the monolayer and assess the partitioning of nAu and G6Pase in the system. Samples for AFM imaging were prepared by transferring monolayers from the Langmuir trough to mica supports. Mixed monolayer films were compressed to 30 mN m^-1^ and transferred to solid mica supports at a vertical transfer rate of 1 mm min^-1^ while maintaining a constant surface pressure. Samples were allowed to dry and imaged by AFM within 24–48 h. The solid-supported monolayers were scanned in air under ambient conditions using a Park Systems XE-100 Advanced Scanning Probe Microscope and XE-Series controller (Park Systems Inc., Santa Clara, CA). Topography and phase contrast images were simultaneously acquired in tapping mode, using silicon probes (Park Systems, PPP-NCHR) with a nominal spring constant of 42 N m^-1^, a resonant frequency of 320–350 kHz, and a tip radius < 10 nm. Images were analyzed using Park Systems XEI software.

### Transmission electron microscope imaging

Transmission electron microscopy imaging was used to provide additional information on the localization of nAu in compressed, mixed monolayer films. Samples were prepared by horizontal Langmuir-Shaefer transfer: 400 mesh copper (Cu) grids with film coatings of both Formvar and carbon (Ladd Research Industries Inc., Williston, VT) were lifted through the compressed monolayer interface on the Langmuir trough. Samples were subsequently dried on filter paper and imaged using a JEOL USA Inc. (Peabody, MA) 2011 Scanning TEM operated at 200 keV and images were captured by a Gatan (Pleasanton, CA) camera.

## Results

### Effects of nAu on membrane monolayer properties

**Fig 1:**
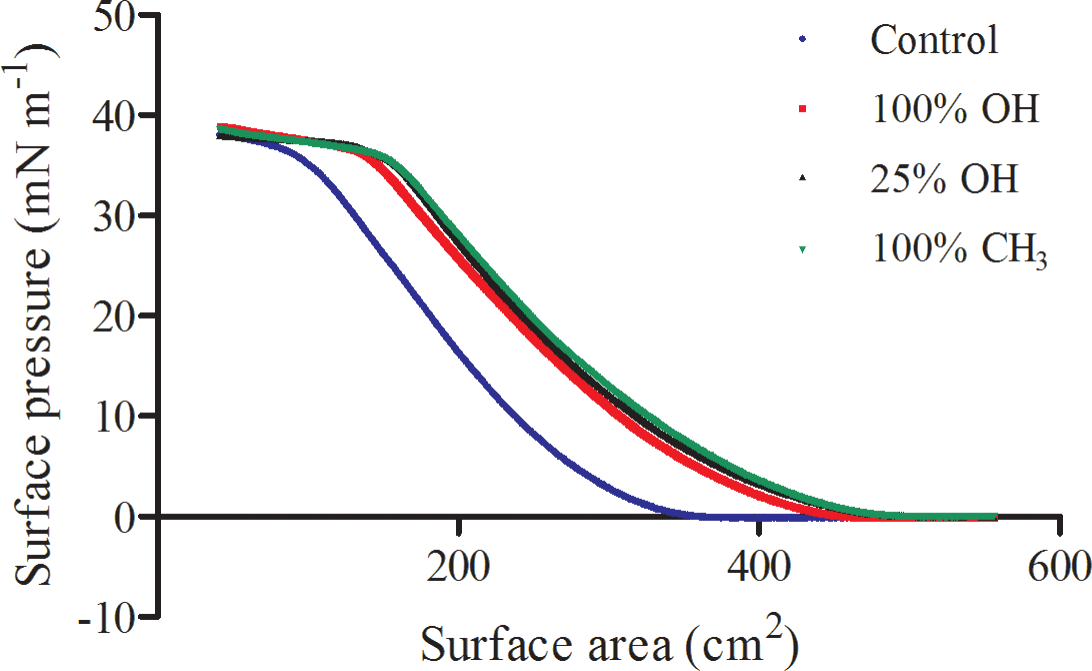
Surface pressure *versus* surface area isotherms of monolayers containing DPPC and G6Pase, in the absence (control) or presence of nAu. Control (blue), nAu functionalized with 100% OH (red), nAu functionalized with 25% OH (black), nAu functionalized with 100% CH_3_ (green).

Representative surface pressure *versus* surface area isotherms of monolayers containing DPPC, G6Pase, and each different nAu formulation are presented in Fig 1, along with a control containing only DPPC and G6Pase. Both the control and nAu-containing films display a broad liquid-expanded (fluid) phase over the compression range, with monolayer collapse at a surface pressure of ~37 mN m^-1^. The horizontal shift between the control and nAu treatments can be accounted for (to within ~15% of the nAu interfacial area per particle measured in pure nAu films [37]) by the interfacial area occupied by the nAu included in these membranes, indicating that the nAu are residing at the air-water interface. The consistent response to compression in terms of slope and collapse pressure indicate that the macroscopic properties (fluidity) of the membrane are not affected by the presence of nAu at this low dosage.

### Partitioning of nAu and G6Pase in the monolayer

**Fig 2:**
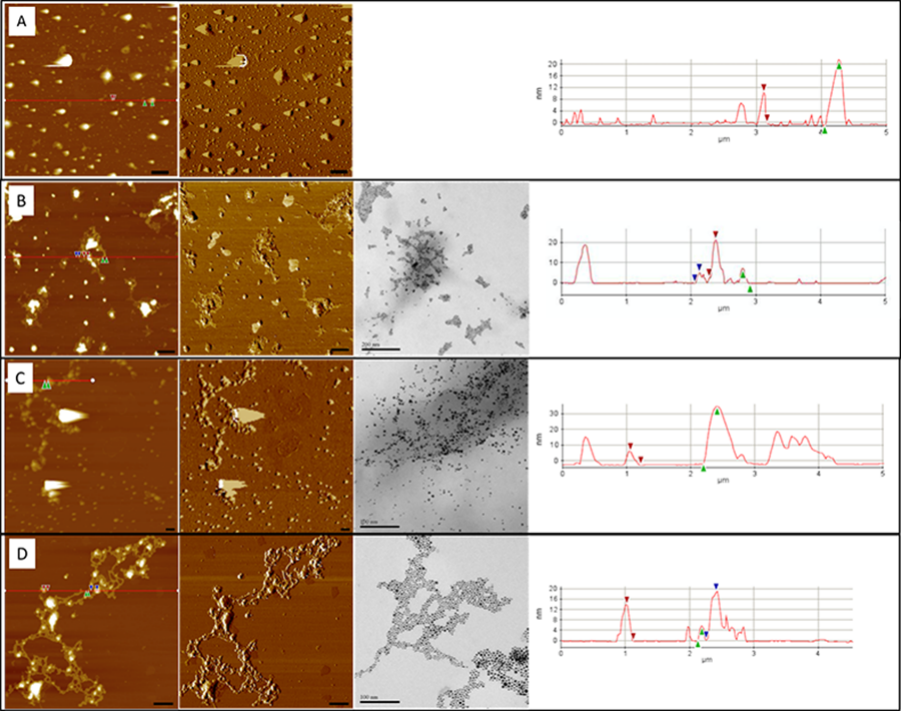
AFM and TEM images of the control (A), 100% CH_3_ (B), 25% OH (C), and 100% OH (D) terminated nAu. Left: AFM topography; middle: corresponding AFM phase; right: TEM images. Note that the TEM images do not correspond to the areas displayed in the AFM images. Line scans on far right correspond to red lines in AFM topography images. Scale bars on topographical and phase images are 500 nm and on TEM images they are 200 nm for 100% CH_3_ terminated nAu (B) and 100 nm for both the 25% OH (C) and 100% OH terminated nAu (D).

Samples of the monolayer films transferred at 30 mN m^-1^ were collected for morphological analysis by both AFM and TEM (Fig 2). AFM topographical images showed that in the absence of nAu, G6Pase formed relatively large aggregates of variable diameters, on average 23 ± 7 nm tall (S2 Table). The dimensions of G6Pase have not been characterized, but the largest dimension of a single protein of similar molecular weight is approximately 14 nm [41]. It is likely that each peak represents several stacked proteins. The AFM phase imaging clearly indicates material contrast of these large features from the flat DPPC monolayer, indicative of protein. Upon addition of nAu, the lateral dimensions of the putative G6Pase protein regions (or aggregates thereof) become less variable, and generally appear to be smaller. In the case of the 100% OH nAu, the height of these protein regions is drastically reduced to 17 ± 4 nm tall. Analysis of the TEM images found core diameters for each nAu formulation of (mean ± SD, N>100) 4.4 ± 0.8 nm for 100% OH, 5.3 ± 1.4 nm for 25% OH, and 4.6 ± 0.9 nm for 100% CH_3_. The interfacial behavior of the nAu used in this study was assessed in an earlier study and revealed that in the absence of other components, the nAu remain pinned to the air/water interface and form close-packed monolayers [37]. AFM topographical images (Fig 2 and S2 Table) displayed regions of closely packed material of 5–6 nm in height matching the TEM size ranges, contrasted against the largely featureless phospholipid background. TEM images confirmed that the nAu formed similar patterns of association in the membrane monolayers, further supporting the conclusion that the 5–6 nm regions observed in topographical images are nAu. In the mixed-membrane monolayers, all nAu formulations examined were primarily associated with other nAu particles and with the regions of G6Pase protein. AFM phase imaging also revealed distinct contrast corresponding to the taller protein regions and the 5 nm tall (nAu) regions, compared to the phospholipid background (S2 Table).

Size analysis of multiple AFM images revealed that co-spreading nAu with the phospholipid and G6Pase components of the monolayer clearly reduced the (lateral) size of the G6Pase protein aggregates in comparison to monolayers prepared without nAu. The magnitude of this effect was dependent upon the ligand shell composition of the nAu and directly related to measurements of G6Pase activity in the membrane (see below). The pattern of nAu partitioning was generally in favor of the protein (vs. the lipid membrane) in the co-spread monolayers and also clearly influenced by differences in the surface functional groups present on the ENMs (Fig 2). The 100% CH_3_ terminated nAu were tightly associated around smaller clusters of protein, while clearly maintaining significant ENM-ENM interactions. The 25% OH terminated nAu formulation induced the greatest dispersal of the G6Pase into smaller aggregates throughout the monolayer and the strongest partitioning toward nAu-protein interactions. Both hydroxyl terminated nAu formulations adopted a web-like pattern around and between protein clusters, however this was more exclusive in the case of 100% OH nAu. Similar to the 100% CH_3_, the 100% OH nAu are involved in nAu-nAu interactions at the air-water interface.

Further investigations into potential direct interactions between nAu and G6Pase were carried out in aqueous suspension. When G6Pase was exposed to a suspension of 100% OH nAu, both tryptophan fluorescence and the hydrodynamic diameter of the protein aggregate significantly decreased by ~8% (P = 0.004 and 0.007, respectively), suggesting some interaction between G6Pase and nAu (S2 Fig).

### Effects of nAu on G6Pase activity

**Fig 3:**
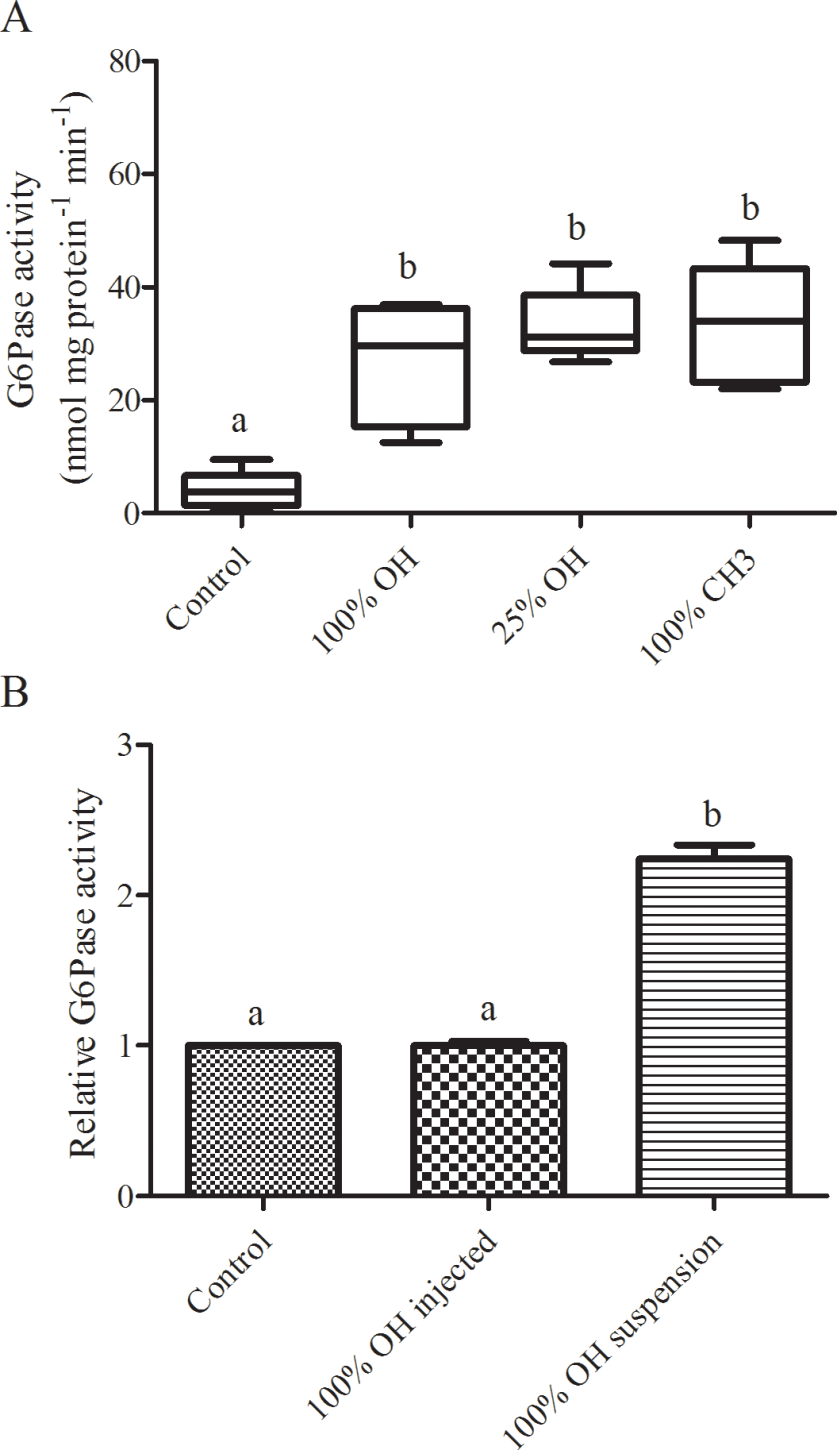
(A) G6Pase activity in a compressed (30 mN m^-1^) mixed monolayer co-spread with or without (control) differentially terminated nAu (N = 6–9). (B) Relative activity (expressed as a proportion of control) of G6Pase in a compressed mixed monolayer with 100% OH terminated nAu injected into the subphase (100% OH injected) and of suspended G6Pase exposed to 100% OH terminated nAu (100% OH suspension; N = 3 for both). Activity is expressed per mg protein preparation in the mixed monolayer. Statistically significant differences are indicated by dissimilar letters.

The effects of differently functionalized nAu on the catalytic activity of G6Pase was quantified in the mixed monolayer under 2 different exposure conditions; with nAu co-spread with DPPC and G6Pase prior to compression, and with nAu injected into the subphase following compression of the monolayer (Fig 3). In co-spread monolayers, all nAu formulations significantly increased G6Pase activity 5–6 fold compared to control monolayers without nAu (P < 0.0001). No significant differences in G6Pase activity were noted between individual nAu formulations. A similar result was also observed when G6Pase activity was assessed in aqueous suspension, where exposure to 100% OH terminated nAu significantly increased activity >2 fold (Fig 3; P = 0.006). This phenomenon was not observed when 100% OH terminated nAu were introduced into the subphase beneath monolayers compressed to 30 mN/m, with no differences in activity noted after a 50 min exposure (P = 0.912).

**Fig 4:**
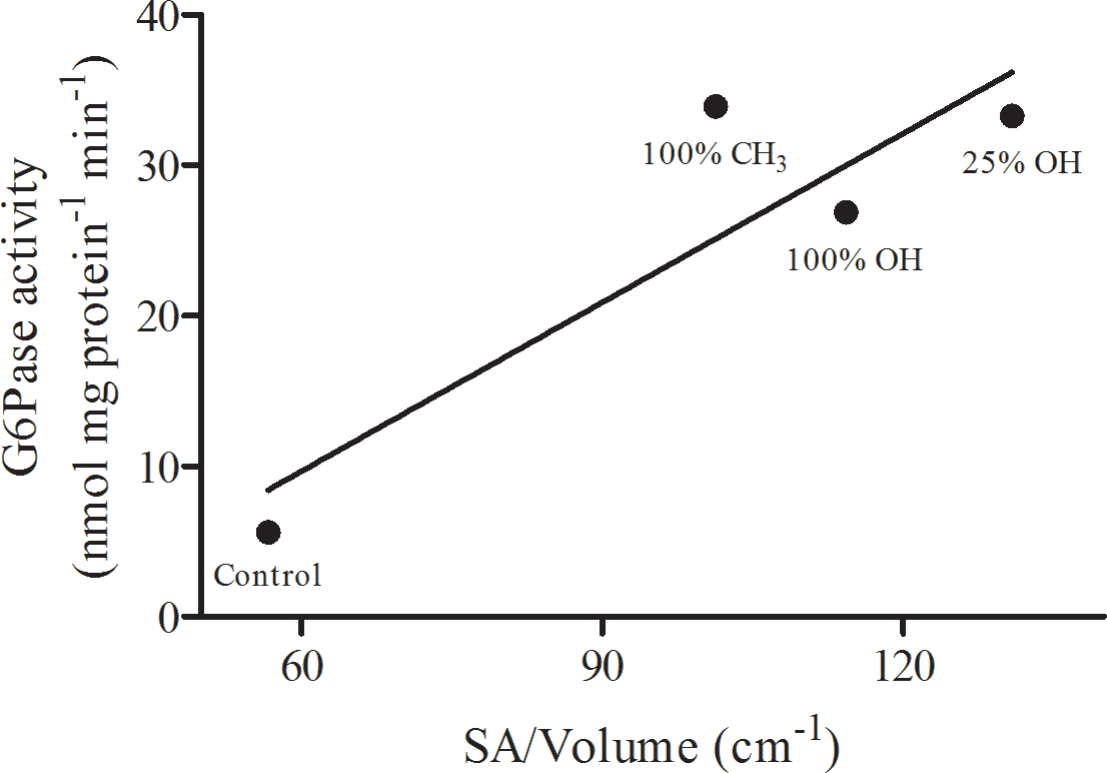
G6Pase activity *versus* protein surface area to volume ratio in compressed (30 mN m^-1^) mixed monolayers co-spread with or without differentially terminated nAu. Activity is expressed per mg protein preparation in the mixed monolayer. Protein surface area to volume ratios in the membrane were determined by AFM. The line of best fit has an R^2^ value of 0.8048.

Grain analysis on AFM images was used to calculate mean surface area to volume ratios (SA:V) of the putative protein aggregations observed in monolayers co-spread with each nAu formulation. This data was then plotted against the measured G6Pase activity under each condition to determine if reductions in the size of G6Pase aggregates associated with nAu may influence catalytic activity in co-spread monolayers (Fig 4). A positive correlation between G6Pase SA:V and activity was observed (R^2^ = 0.8048), with the SA:V of G6Pase aggregates generally increasing with increasing nAu hydrophilicity. Although the slope of the regression line was not significantly different from 0 (P = 0.103), this data supports qualitative observations of AFM images and suggests that nAu interact with G6Pase in a formulation-specific manner.

## Discussion

The aim of this study was to characterize the partitioning of hydrophobic nAu in a mixed protein-phospholipid monolayer and to understand whether they impact membrane protein function differently *in situ*. G6Pase, an integral membrane protein, was successfully incorporated into DPPC monolayers and catalytic activity was maintained at physiological membrane pressures. In aqueous suspension, tryptophan fluorescence and dynamic light scattering data showed that nAu-G6Pase interactions did occur, and catalytic activity increased as a result. When co-spread with phospholipid and protein in a monolayer and compressed to physiological pressures, nAu indeed increased the catalytic activity regardless of nAu composition. Analysis of the membranes by AFM and TEM indicate that the nAu generally partitioned to the protein, rather than distributing throughout the phospholipid membrane. This partitioning affected the distribution of G6Pase within the membrane, ostensibly decreasing the size of protein aggregates and promoting catalytic activity. These effects were not observed in acute studies where nAu were introduced through the subphase below compressed membranes, although specific cellular conditions may promote ENM incorporation (see below). Varying the relative hydrophobicity of surface functional groups on nAu with identical core structures altered the pattern of nAu partitioning in co-spread monolayers, indicating that the physicochemical properties of the ENM may influence membrane bioactivity differentially with composition and dosage.

### nAu affects membrane protein distribution

In the absence of nAu, G6Pase did not homogeneously distribute throughout the membrane, forming large protein aggregates up to several hundred nm in diameter (Fig 2A). In membranes co-spread with nAu, AFM images illustrated close associations between nAu and protein regions, suggesting all three nAu formulations preferentially associated with protein and each other, rather than dispersing into the phospholipid matrix. A key finding is that nAu-nAu interactions compete with nAu-protein interactions in the most and least hydrophilic formulations and that partitioning in the intermediate 25% OH nAu is dominated by nAu-protein interactions. In TEM images, the nAu also remain primarily at the air-water interface, as opposed to decorating the protein aggregates throughout. These data illustrate that even amongst hydrophobic ENMs, altering the relative hydrophobicity of a surface functional group can substantially influence particle behavior in a biological system. Furthermore, these results suggest that nAu are only partially associated with G6Pase in solution prior to spreading and that much of the partitioning is indeed occurring within the membrane. Thus, while hydrophobic ENMs likely have fewer mechanisms by which they can be delivered to biological membranes, they can lead to some restructuring of the membrane once incorporated.

Interactions between nAu and protein influenced protein-protein interactions, reducing the size of G6Pase aggregates observed in the AFM topographical images (Fig 2). Surface area to volume ratios of G6Pase aggregates were >2 fold higher in membranes co-spread with any of the 3 nAu formulations studied here (Fig 4). Aggregation often results from interactions between exposed hydrophobic amino acid residues on neighboring proteins in the aqueous cellular environment [42]. In this case, G6Pase aggregates were likely formed during the protein extraction and purification process, where hydrophobic membrane spanning domains on adjacent G6Pase subunits were brought into close association as the protein was concentrated. The partitioning data suggest that nAu-protein binding was more thermodynamically favorable than these protein-protein interactions, leading to a reduction in the observed size of G6Pase aggregates in the membrane. In aqueous suspension, the hydrodynamic diameter of G6Pase was reduced in the presence of 100% OH terminated nAu, however this reduction is not enough to account for the >2-fold increase in SA:V observed in the monolayer. It is most likely that nAu-protein interactions limited the size of G6Pase aggregates in suspension prior to spreading, and further prevented the propagation of aggregates during monolayer formation, therefore improving “solubilization” of the protein in the membrane.

### nAu likely affects protein function indirectly

The smaller size of G6Pase aggregates in the presence of nAu was strongly associated with increases in the catalytic efficiency of the protein, both in compressed membranes and in aqueous suspension. G6Pase activity was 5 to 6-fold higher in membranes co-spread with any of the three nAu formulations tested and >2-fold higher when exposed to 100% OH terminated nAu in suspension. By reducing the size of G6Pase aggregates, nAu-protein interactions likely increased the number of accessible active sites and facilitated catalysis. An alternative explanation is direct interactions with nAu altered the native structure of G6Pase, allosterically activating the protein. ENMs can change protein structure [8,10,40] and the modest quenching of G6Pase tryptophan fluorescence observed here (Fig 3) indicates structural changes may have occurred. Given that such changes were minor compared to the >2-fold change in the surface area to volume ratios of protein aggregates, increases in activity arising from structural changes are likely insignificant.

The influence of relative surface functional group hydrophobicity on membrane protein function remains unclear. While all 3 nAu formulations tested solubilized G6Pase aggregates, the intermediate 25% OH terminated nAu were the most effective. Given the distinct properties of the CH_3_ and OH-terminated nAu, it is likely they form favorable interactions with the protein by different mechanisms. If this is the case, the 25% OH nAu may engage in both mechanisms to optimally interact with G6Pase to reduce aggregate size and promote activity. The modest differences in bioactivity between nAu formulations in terms of both solubilization and catalytic activity suggests disaggregation is not likely an equilibrium process, but rather is limited to the period of spreading and evaporation of solvents used during monolayer formation. Given the unexpectedly large solubilization effect observed here, it is difficult to discern subtle differences in functional effects the ENMs may be having on the protein. Testing a broader range of nAu formulations, dosages, or different phospholipid components may also provide insight into the relative contribution of nAu-protein interactions and nAu-induced changes in local membrane properties (viscosity, elasticity, etc.) to protein function. The most hydrophilic nAu formulation (100% OH terminated) formed web-like patterns throughout the membrane, effectively stringing G6Pase aggregates together, while 100% CH_3_ terminated nAu clustered tightly around protein aggregates (Fig 2). Changes in membrane properties can influence the catalytic efficiency of membrane proteins [43,44] and changes in protein distribution may have repercussions on biological function, so such differences in ENM partitioning between protein, other ENM, and phospholipid could influence protein function by different mechanisms.

In a similar monolayer system (without protein), graphene oxide ENMs partitioned between phospholipid molecules when co-spread prior to compression [27]. The ability of the ENM to incorporate into the membrane was highly dependent upon the charge of the phospholipid head group, with positively charged phospholipids accommodating the complimentary negative charge of the graphene oxide [27]. The net neutral charge of DPPC’s head group may explain why clear links were not observed between nAu-protein/lipid interactions and relative nAu hydrophobicity. All nAu formulations tested preferentially partitioned to the protein rather than localizing to the acyl chains or zwitterionic head group of DPPC. In a biological membrane expressing a range of heterogeneously distributed lipid classes, cholesterol, and peripheral and integral membrane proteins, ENM partitioning will be much more complex.

### Surface pressure inhibits acute nAu incorporation

In the current work, 100% OH terminated nAu did not influence G6Pase activity when introduced through the subphase (Fig 3), indicating they were unable to penetrate the compressed membrane over the short time scale of the experiment (60 min). This result agrees with the results obtained from the AFM imaging, which suggests that while the nAu-protein interactions are significant, they do not strongly out-compete nAu-nAu or nAu-water interactions. Stronger nAu-protein interactions are likely necessary to observe nAu penetration into a compressed membrane. Li et al. [27] showed that graphene oxide was able to incorporate into some pre-compressed phospholipid monolayers, but not others, and that incorporation depended upon the nature of the lipid head group. The high surface pressure used in the current study (30 mN m^-1^) may prevent interactions between nAu and the hydrophobic domains of G6Pase that are necessary to facilitate nAu incorporation into the membrane. When closely associated in the compressed monolayer, DPPC’s head groups may also shield interactions between nAu and the protein. Incorporation might occur over longer exposure durations, higher ENM concentrations, or lower surface pressures but further study is necessary to confirm this possibility.

### Implications for bioactivity and toxicity

Lipid composition differs greatly between the ER and plasma membranes, with the former having mostly loosely packed neutral lipids like DPPC and the latter tightly packed anionic lipids [45]. The non-polar CH_3_ terminated nAu studied here may therefore exhibit greater bioactivity at the plasma membrane relative to the negatively charged OH terminated nAu, which would interact more favorably with intracellular membranes such as the ER. The phagocytosis of extracellular ENMs and subsequent release from damaged lysosomes represents one potential route of intracellular ENM exposure [46]. The highly dynamic nature of biological membranes *in vivo* may also present circumstances where energetic barriers to ENM incorporation are reduced, such as fluctuations in surface pressure associated with acute temperature change or cellular stress.

ENMs can directly or indirectly damage lipid bilayers [47] and cause protein misfolding [48], leading to toxicity in clinical applications and in the environment. Understanding the core mechanisms underlying ENM partitioning in mixed membrane systems, like that employed here, will allow us to better predict the toxicity of novel ENMs and improve the targeting and efficacy of nano-enabled pharmaceuticals.

## Supporting information

S1 Fig Sodium dodecyl sulfate-polyacrylamide gel electrophoresis (SDS-PAGE) of the crude G6Pase preparation utilized in the study. 3 μg of the G6Pase preparation in Laemmli buffer was loaded into a Mini-Protean TGX gel (Bio-Rad, Hercules CA, USA) and electrophoresed in Tris running buffer at a constant 200 V. The gel was stained for total protein (SimplyBlue SafeStain, Invitrogen, Carlsbad, CA, USA). A molecular weight (in kiloDaltons, kDa) ladder (Precision Plus, Bio-Rad) is included for comparison.
(TIF)

S2 Fig **The effects of 100% OH terminated nAu on (A) fluorescence of tryptophan residues in G6Pase (in relative fluorescence units, RFU; N = 4 for both treatments) and (B) the hydrodynamic diameter of G6Pase in suspension (N = 3 for both treatments).** Asterisk indicates significant difference from control.
(TIF)

S1 Table Profile of fatty acid methyl esters (FAME) in the lipid component of the purified G6Pase sample. (DOCX)

S2 Table Atomic force microscopy height and phase measurements (relative to DPPC monolayer) of regions in mixed monolayers putatively labeled as protein or nAu regions. Data are presented as mean ± standard deviation.
(DOCX)
