# Supplementary figures and images for: Gold nanoparticles partition to and increase the activity of glucose-6-phosphatase in a synthetic phospholipid membrane system
Source: PLoS One. 2017 Aug 17;12(8):e0183274. doi: 10.1371/journal.pone.0183274 (PMC5560555; doi:10.1371/journal.pone.0183274)

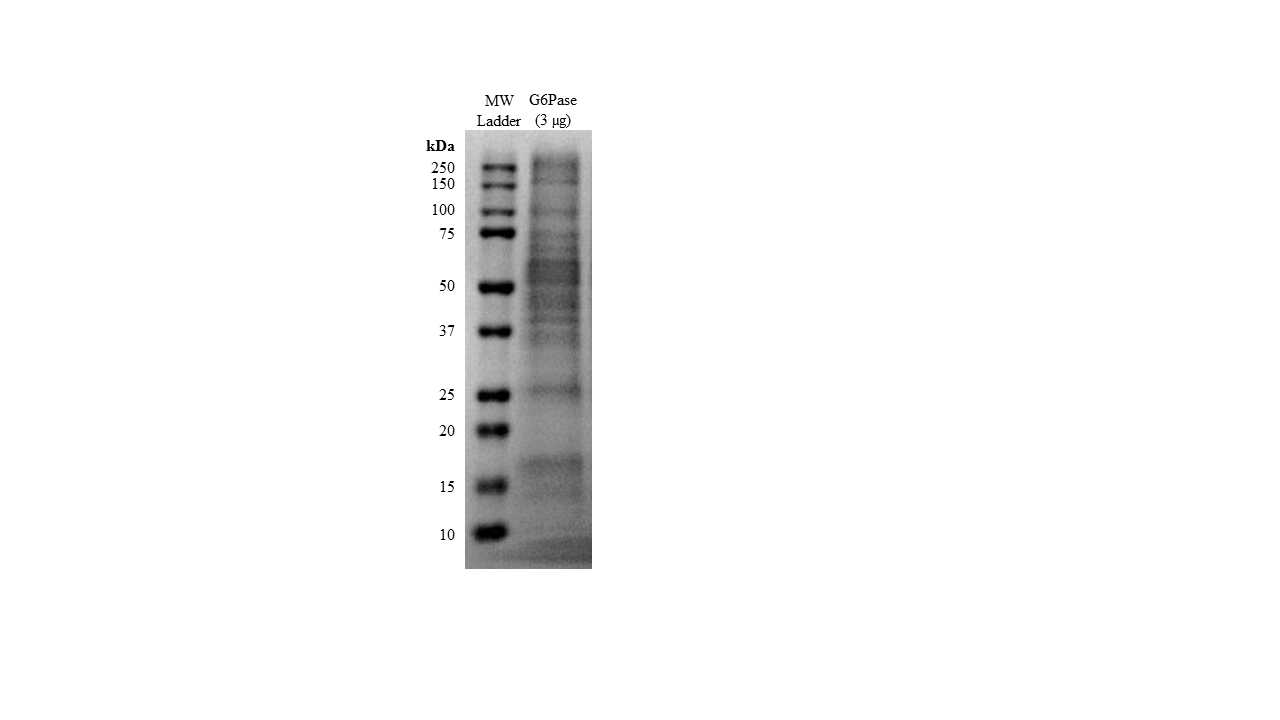

Supplement: S1 Fig — 3 μg of the G6Pase preparation in Laemmli buffer was loaded into a Mini-Protean TGX gel (Bio-Rad, Hercules CA, USA) and electrophoresed in Tris running buffer at a constant 200 V. The gel was stained for total protein (SimplyBlue SafeStain, Invitrogen, Carlsbad, CA, USA). A molecular weight (in kiloDaltons, kDa) ladder (Precision Plus, Bio-Rad) is included for comparison. (TIF) [file pone.0183274.s001.tif]

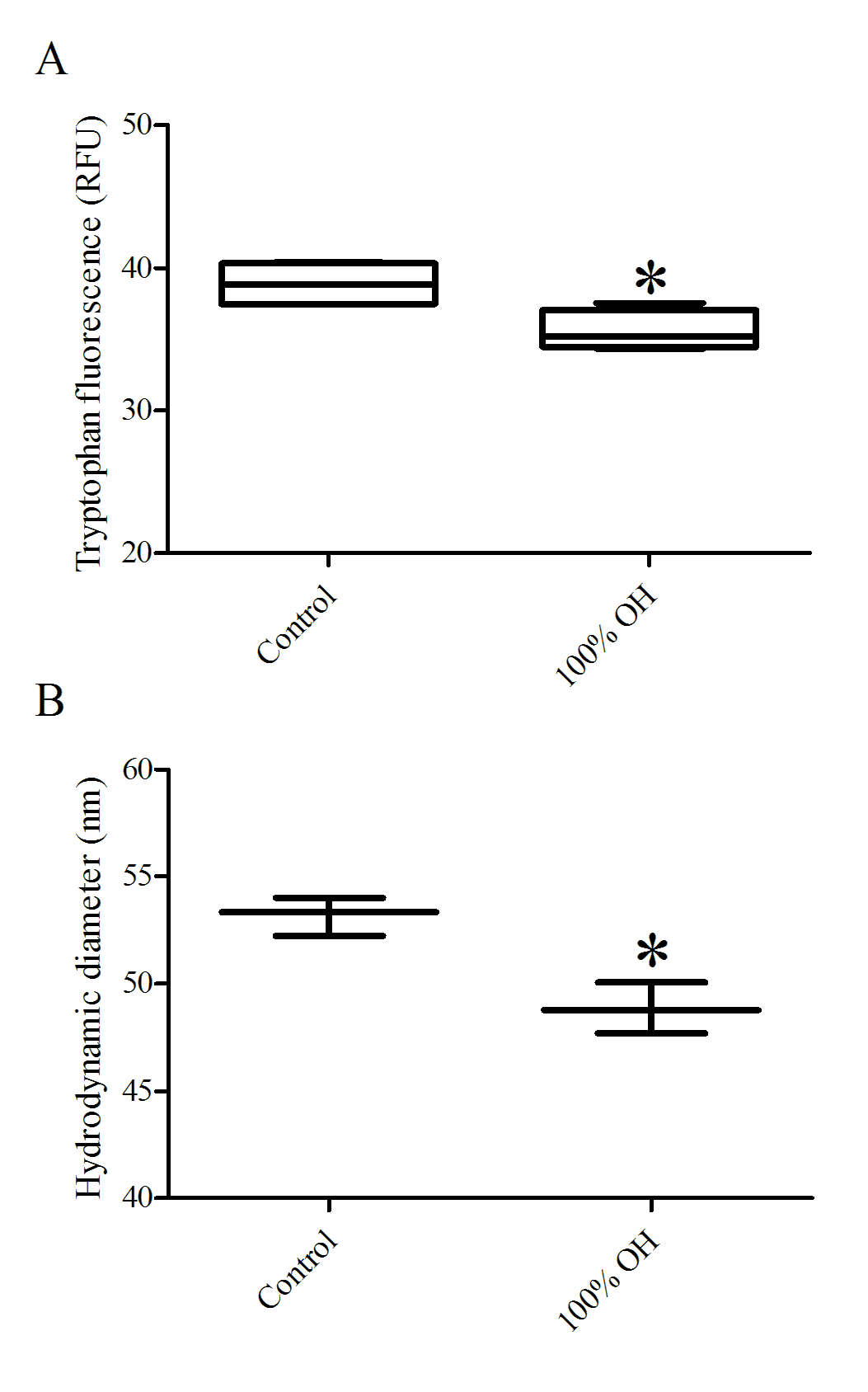

Supplement: S2 Fig — The effects of 100% OH terminated nAu on (A) fluorescence of tryptophan residues in G6Pase (in relative fluorescence units, RFU; N = 4 for both treatments) and (B) the hydrodynamic diameter of G6Pase in suspension (N = 3 for both treatments). Asterisk indicates significant difference from control. (TIF) [file pone.0183274.s002.tif]
