# Supplementary material for: Gold nanoparticles partition to and increase the activity of glucose-6-phosphatase in a synthetic phospholipid membrane system
Source: PLoS One. 2017 Aug 17;12(8):e0183274. doi: 10.1371/journal.pone.0183274 (PMC5560555; doi:10.1371/journal.pone.0183274)
Supplement: S1 Table — (DOCX) [file pone.0183274.s003.docx]

| FAME | ug FAME/mg | nmol FAME/mg | % FAME (ug/mg) | % FAME (nmol/mg) |
| --- | --- | --- | --- | --- |
| 14:0 | 0.22 | 0.92 | 0.86 | 1.03 |
| 14:1n-5 | 0.00 | 0.00 | 0.00 | 0.00 |
| 16:0 | 8.47 | 31.37 | 32.91 | 35.23 |
| 16:1n-7 | 0.14 | 0.51 | 0.53 | 0.57 |
| 18:0 | 7.57 | 25.40 | 29.41 | 28.53 |
| 18:1n-9 | 3.06 | 10.35 | 11.90 | 11.62 |
| 18:1n-7 | 0.24 | 0.80 | 0.92 | 0.90 |
| 18:2n-6 | 4.05 | 13.76 | 15.72 | 15.45 |
| 18:3n-6 | 0.00 | 0.00 | 0.00 | 0.00 |
| 18:3n-3 | 0.11 | 0.38 | 0.43 | 0.42 |
| 20:0 | 0.35 | 1.06 | 1.34 | 1.19 |
| 18:4n-3 (SDA) | 0.00 | 0.00 | 0.00 | 0.00 |
| 20:1n-9 | 0.05 | 0.16 | 0.21 | 0.18 |
| 20:2n-6 | 0.07 | 0.21 | 0.26 | 0.23 |
| 20:3n-6 | 0.10 | 0.30 | 0.38 | 0.34 |
| 20:4n-6 (AA) | 0.46 | 1.46 | 1.80 | 1.64 |
| 20:3n-3 | 0.00 | 0.00 | 0.00 | 0.00 |
| 22:0 | 0.38 | 1.08 | 1.49 | 1.22 |
| 22:1n-9 | 0.05 | 0.15 | 0.21 | 0.17 |
| 20:5n-3 (EPA) | 0.00 | 0.00 | 0.00 | 0.00 |
| 22:4n-6 | 0.05 | 0.16 | 0.21 | 0.18 |
| 24:0 + 22:5n-6 | 0.37 | 0.96 | 1.43 | 1.08 |
| 24:1n-9 | 0.00 | 0.00 | 0.00 | 0.00 |
| 22:5n-3 (DPA) | 0.00 | 0.00 | 0.00 | 0.00 |
| 22:6n-3 (DHA) | 0.00 | 0.00 | 0.00 | 0.00 |
| *Total:* | 25.7 | 89.0 | 100.0 | 100.0 |
