# Supplementary material for: Gold nanoparticles partition to and increase the activity of glucose-6-phosphatase in a synthetic phospholipid membrane system
Source: PLoS One. 2017 Aug 17;12(8):e0183274. doi: 10.1371/journal.pone.0183274 (PMC5560555; doi:10.1371/journal.pone.0183274)
Supplement: S2 Table — Data are presented as mean ± standard deviation. (DOCX) [file pone.0183274.s004.docx]

|  |  | Control  (Lipid + Protein) | 100% OH | 25% OH | 100% CH_3_ |
| --- | --- | --- | --- | --- | --- |
| Protein | Height (nm) | 23.1 ± 6.5 | 16.7 ± 3.8 | 26.0 ± 2.6 | 28.6 ± 13.4 |
|  | Phase Shift (°) | 4.5 ± 0.3 | 4.7 ± 0.3 | 5.2 ± 1.4 | 5.4 ± 0.9 |
| nAu | Height (nm) | -- | 4.6 ± 0.6 | 6.2 ± 1.1 | 4.5 ± 1.9 |
|  | Phase Shift (°) | -- | 3.5 ± 0.5 | 3.2 ± 1.3 | 4.3 ± 0.4 |
